# Imaging as a biomarker in drug discovery for Alzheimer’s disease: is MRI a suitable technology?

**DOI:** 10.1186/alzrt276

**Published:** 2014-07-30

**Authors:** Emilio Merlo Pich, Andreas Jeromin, Giovanni B Frisoni, Derek Hill, Andrew Lockhart, Mark E Schmidt, Martin R Turner, Stefania Mondello, William Z Potter

**Affiliations:** 1 Clinical Imaging, Neuroscience DTA pRED, F. Hoffman-La Roche, Grenzacherstrasse 124 CH-4070, Basel, CH, Switzerland; 2 Atlantic Biomarkers, LLC, 316 NW 28th Terrace, Gainesville, FL 32607, USA; 3 IRCCS San Giovanni di Dio Fatebenefratelli, Laboratory of Epidemiology, Neuroimaging, and Telemedicine, via Pilastroni 4, Brescia 25125, Italy; 4 Medical Imaging Science, UCL, London, UK; 5 IXICO Ltd, Floor 4, Griffin Court, 15 Long Lane, London EC1A 9PN, UK; 6 GlaxoSmithKline, Neurodegeneration DPU R&D China, Neurosciences TA Unit, Clinical Unit Cambridge, Addenbrookes Hospital, Cambridge CB2 2GG, UK; 7 Experimental Medicine, Neuroscience Therapeutic Area, Janssen Pharmaceutica NV, Turnhoutseweg 30, B-2340, Beerse 2340, Belgium; 8 Oxford University Nuffield, Department of Clinical Neurosciences, John Radcliffe Hospital, Oxford OX3 9DU, UK; 9 Department of Neurosciences, University of Messina, Via Consolare Valeria, 98125 Messina, Italy; 10 National Institute of Mental Health, 6001 Executive Boulevard, BG NSC RM 7209, Rockville, MD 20892, USA

## Abstract

This review provides perspectives on the utility of magnetic resonance imaging (MRI) as a neuroimaging approach in the development of novel treatments for Alzheimer’s disease. These considerations were generated in a roundtable at a recent Wellcome Trust meeting that included experts from academia and industry. It was agreed that MRI, either structural or functional, could be used as a diagnostic, for assessing worsening of disease status, for monitoring vascular pathology, and for stratifying clinical trial populations. It was agreed also that MRI implementation is in its infancy, requiring more evidence of association with the disease states, test-retest data, better standardization across multiple clinical sites, and application in multimodal approaches which include other imaging technologies, such as positron emission tomography, electroencephalography, and magnetoencephalography.

## Introduction

This article is the result of a roundtable held at the Wellcome Trust in the context of the meeting 'Biomarkers for Brain Disorders: Challenges and Opportunities’ in February 2013. The focus was on the relevance of neuroimaging biomarkers in the development of novel treatment in Alzheimer’s disease (AD), with a particular focus on magnetic resonance imaging (MRI). AD is the most common form of senile dementia associated with cognitive decline, regional brain hypometabolism/atrophia, β-amyloid plaque deposition, accumulation of phosphorylated tau-containing neurofilament tangles (NFTs), vascular pathology, and neuroinflammation, and the diagnostic criteria of AD were recently amended [1]. Based on the experience of current precompetitive consortia such as the Alzheimer Disease Neuroimaging Initiative (ADNI) [2], the Innovative Medicine Initiative Pharma-Cog [3], or the Dominant Inherited Alzheimer Network [4], the general consensus was that MRI has great potential for improving novel drug development but is not a mature biomarker yet. As for other biomarkers, the current applications of neuroimaging include assistance in (a) the diagnosis of dementia, (b) the selection of subjects for enrollment in clinical trials or for stratification, (c) the tracking of disease progression, (d) providing evidence for target engagement for new therapeutic agents, and (e) providing evidence of disease-modifying effects (that is, normalization of a pathologic signal in association with clinical improvement (surrogate marker)). The MRI literature is partially supportive for the first three applications, whereas the database for the last two applications is still growing [5-7]. The present article should not be seen as an exhaustive review of neuroimaging in AD research (as shown in [8-10]) but as an introduction to the uneven status of advancement of MRI technology for drug development.

## A practical question

Developing a new drug is an evidence-based exercise. Collecting evidence relevant for decision-making to support the development of a novel chemical entity (NCE) requires investment in methodologies that are reliable, valid, and clinically relevant. In other words, we should answer the question: is it worthwhile to include a neuroimaging assessment in a clinical trial for a novel treatment in AD? If the method has regulatory approval, the inclusion in a clinical trial is generally supported. If not, the quality of the scientific data, the simplicity in execution, the reliability in multi-center assessment, and a reasonable cost would drive the decision for including imaging endpoints. In this case, expectations about trial outcome should be adjusted since the imaging measurements may deliver exploratory data whose real value will be appreciated only when combined with data coming from other research groups, possibly organized within precompetitive consortia (for example, ADNI). Remarkably, only a limited number of imaging biomarkers are endorsed by regulatory agencies (Table 1). A series of methodological challenges are still facing the neuroimaging scientific community. The most important are (a) the interpretation of the biological meaning of the various neuroimaging measurements and their relationship with disease severity, (b) the test-retest reliability, and (c) the proper implementation into multi-center clinical trials. Hence, one of the main goals of the scientific community should be to provide answers to these questions and contribute to the regulatory approval of the neuroimaging methods.

**Table 1:** Evidentiary table showing different imaging modalities implemented in AD research with relevant information

	**Patient population**	**Current use. (Potential clinical use)**	**Cost; feasibility. Burden**	**Biological and multi-center validation**	**Limitation**	**Regulatory status**
FDG PET	MCI, AD, and other dementias	Diagnostic; stratification [10]; provide support to therapeutic clinic effects [11]. (Disease progression.)	High cost; - Feasible. - IV infusion radioactive agent needed.	Biological post-mortem data. Guidelines for single- and multi-site use, vendor-specific protocols. Standardized for multi-center trials. High validity [12].	Signal affected by inflammation and ischemia or behavior state [9,13]; misdiagnosis reported [14].	Indicated for differential diagnosis of dementia (FDA and EMA) in 2004. No regulatory qualification for use in early AD at this time.
Amyloid-β PET	MCI, AD, and other dementias	Diagnostic: AD exclusion; stratification; target engagement [15].	High cost; - Difficult. - IV infusion radioactive agent needed.	Biological post-mortem data [16]. Guidelines for single- and multi-site use, vendor-specific protocols. Standardization for multi-center trials partially achieved [12].	Cases of normal subjects with high amyloid-β and cases of subjects with dementia with low amyloid-β. Detection threshold to be validated [8].	Indicated for differential diagnosis of dementia in 2012 (FDA and EMA). Qualified as baseline measure for selecting patients for trials in pre-dementia and mild-moderate AD (EMA). Regulatory guidance for use in research and drug development.
Volumetric MRI	MCI, AD	Diagnostic [10]; stratification using hippocampal volume [17,18]. (Disease progression; provide support to therapeutic clinic effects.)	Low cost; - Easy. Burden: no pacemaker carriers; claustrophobic reaction.	SOPs for single and multi-site; vendor-specific protocols. Standardization for multi-center trials partially achieved [6,9].	Lack molecular specificity. Direct data on hippocampal histopathology. Loss of volume as non-specific atrophy [9]. Patterns overlap with non-AD disease or in advanced aging.	Hippocampal volume qualified as baseline measure for selecting patients for trials in pre-dementia AD/MCI through EMA. Hippocampal volume qualification for patient stratification in MCI/AD through CAMD/FDA in progress.
ARIA MRI	MCI, AD	Safety signal; vascular integrity; inflammation [14].	Low cost; - Easy. Burden: no pacemaker carriers; claustrophobic reaction.	SOPs for single and multi-site; vendor-specific protocols. Standardization for multi-center trials achieved [19].	Occurrence of spontaneous ARIA in elderly unknown. False positive in untreated APP duplication case.	Requirement of monitoring (several MRI scans) during trials with amyloid-β-lowering agents (FDA and EMA).
DTI tracto-graphy	MCI, AD, non-clinical at genetic risk subject	Diagnostic [20]; stratification; disease progression. (Provide support to therapeutic clinic effects.)	Low cost; - Easy. Burden: no pacemaker carriers; claustrophobic reaction.	Biological validation in progress [21]. SOPs for single and multi-site; vendor-specific test protocols; standardization for multi-center trials in progress [22,23].	Head motion artifacts; partial volume effects. Limited direct data on histopathology. Few multimodal relationships established.	No regulatory guidelines
Resting-state fMRI	MCI, AD, non-clinical at genetic risk subject	(Provide support to therapeutic clinic effect [24,25]. Stratification [26]; disease progression [7].)	Low cost; - Easy. Burden: no pacemaker carriers; claustrophobic reaction.	Various SOPs for BOLD in single- [27] and multi-center; ASL in single center [20,28]. Test-retest done [7,29]. Standardization for multi-center trials in progress [26].	Head motion artifacts; unclear biology; few data in histopathology. Preliminary data longitudinal and multimodal data available [7].	No regulatory guidelines
Memory task - fMRI	MCI, AD, non-clinical at genetic risk subject	(Provide support to therapeutic clinic effect. Stratification [24]; disease progression [30].)	Middle cost; - Complex. Burden: no pacemaker; claustrophobic reaction.	Various SOPs for single center [31,32]. Vendor-specific test protocols. No standard for multi-center trials. Test-retest not always available [33].	Head motion artifacts; difficult implementation in cognitively impaired subject Longitudinal data available in part; incomplete reliability data [9,34].	No regulatory guidelines

AD, Alzheimer’s disease; APP, amyloid precursor protein; ARIA, amyloid-related imaging abnormality; ASL, arterial spin labeling; BOLD, brain oxygen level-dependent; CAMD, coalition against major diseases; DTI, diffusion tensor imaging; EMA, European Medicines Agency; FDA, US Food and Drug Administration; FDG, fluoro-2-deoxy-D-glucose; fMRI, functional magnetic resonance imaging; IV, intravenous; MCI, mild cognitive impairment; MRI, magnetic resonance imaging; PET, positron emission tomography; SOP, standard operating procedure.

## Neuroimaging in Alzheimer’s disease: the legacy of positron emission tomography

Different neuroimaging modalities are at different stages of maturity regarding implementation in AD research. For example, for more than two decades, molecular neuroimaging assessed with positron emission tomography (PET), aimed to investigate key neurochemical processes at work in AD, has been used with a certain success to assist the diagnostic process in AD research [9,10]. PET with [35]-^18^Fluoro-2-deoxy-D-glucose (FDG) used to map the cerebral metabolic rate of glucose was the first imaging methodology to receive regulatory approval for the diagnostic evaluation of dementia [10,36]. The extent and severity of glucose hypometabolism have been reported to be predictive of conversion to AD in prodromal AD patients, and to correlate with cognitive impairment in prodromal and probable AD [11,12,36]. However, for longitudinal use, careful control and standardization of acquisition are necessary. In fact, as a measure of brain activity, FDG PET is susceptible to biological variability due to normal aging, current state of arousal level, local inflammation, and other non-neuronal sources of metabolic changes, possibly resulting in misdiagnosis [10,13,14].

PET-mediated visualization of the amyloid plaques in the brain was obtained by using amyloid-β-selective radioligands, at first with the Pittsburgh compound B (11C-PiB) and later with the ^18^ F-labeled tracers (for example, florbetapir, flutemetamol, and florbetaben) [16,37]. Since 2011, several of these tracers received regulatory approval to assist in the diagnosis of dementia (in particular, for ruling out AD in favor of other forms of dementia when the amyloid-β signal was absent in the presence of cognitive impairment, in line with the revised Research Diagnostic Criteria for AD) [1]. However, since elevated amyloid load was described in up to 15% of non-demented subjects with age-adjusted normal cognitive profile, it is unclear whether this represents an early marker for individuals that will develop the disease soon or normal variations of a biological parameter with loose association with the disease [35]. More recently, the abundant NFT pathology, another hallmark of AD, has been targeted with the initial development of two novel PET ligands in clinical exploration: 18 F-T-807 and 11C-PBB-3 [38,39]. It is notable that both amyloid-β peptides and phosphorylated tau levels can be measured biochemically in the cerebrospinal fluid and are validated biomarkers in AD research [40], questioning the need for the more expensive neuroimaging.

Amyloid-β PET recently qualified for the European regulatory agency (European Medicines Agency, or EMA) as a biomarker for population enrichment of clinical trials with prodromal and mild-moderate AD subjects (EMA/CHMP/SAWP/893622/2011 and EMA/CHMP/SAWP/892998/2011). Amyloid-β-selective radioligands were also used to provide evidence of target engagement for NCE aimed at amyloid-β plaques (that is, bapineuzumab [19] and gantenerumab [15]). These studies showed a significant dose-dependent reduction of the amyloid-β PET signal over a period of several months of treatment. However, data available from large clinical trials indicate that changes of amyloid-β signal were not associated with changes of disease clinical severity in patients with AD [19,41] and, to date, should not be used as a surrogate marker for efficacy. A final note: the implementation of PET requires exposure to radioactivity that limits the repeated use in the same individuals as well as specialized centers and significant investment. Therefore, while PET can provide unique and useful information, these limitations challenge use in large clinical trials and even in community-based health care. In fact, for these reasons, PET is often implemented in a sub-study of a larger multi-center clinical trial, targeting a sub-population of the whole study.

## Magnetic resonance imaging implementation in Alzheimer’s disease research

MRI-related techniques are gaining increasing interest since their use does not require radioactivity exposure and they can be easily repeated in the same subjects with no harm, are relatively inexpensive, and can be operated with scanner machines available in almost any hospital [6,9,10]. In clinical trials, MRI is commonly used as a radiologic diagnostic aid to exclude individuals with incidental brain pathologies. The evidence supporting their use as specific biomarkers for AD is still limited (Table 1). We briefly describe the most promising use of MRI.

### Structural magnetic resonance imaging

Structural MRI is an imaging modality that describes the shape, size, and integrity of gray and white matter structures in the brain; it is highly sensitive to the atrophic and vascular changes that occur in the AD central nervous system [6].

#### Volumetric magnetic resonance imaging

Volumetric MRI is based on data collected by using T1-weighted sequences that are quantified by using both manual and automatic image analysis. Whereas harmonization of manually segmented hippocampus is in progress [17], automated image analysis software (NeuroQuant; CorTechs Labs Inc., San Diego, CA, USA) was recently approved as a medical device to provide an aid in dementia diagnosis [18].

Volumetric MRI collected longitudinally at two or more time points is the most mature imaging biomarker of disease progression in AD [6], recently supported by multimodal evidence using FDG [36]. Progression of atrophy in the whole brain and in areas targeted by AD (medial temporal lobe, temporoparietal, and restrosplenial cortex) represents a reliable marker of the neurodegenerative process underlying clinical symptoms, more robust than amyloidosis alone. Hippocampal volumetric MRI has been validated versus pathologic post-mortem markers such as neuronal loss and Braak stages [42]. Hippocampal volumetric MRI is qualified by EMA as a biomarker for enrichment of pre-dementia AD trials [17], these prodromal subjects having already lost about 20% of its volume. Progression of whole-brain and hippocampal atrophy has been shown to correlate closely with clinical worsening in patients with AD [6].

Notably, an unexpected paradoxical finding was reported in anti-amyloid immunotherapeutic studies in AD [19,43], showing a reduced volume in hippocampus, whole brain, and cortex. These results are calling for a re-evaluation of the neurobiological interpretation of the volumetric MRI reduction. The short-term reduction of brain volume has been hypothesized to result from changes in fluid balance or amyloid plaque removal rather than a sustained increase in rate of atrophy. Multimodal studies including FDG PET and functional MRI (fMRI) as well as post-mortem studies would eventually lead to a better understanding of the biological underpinning of this phenomenon.

Methodologically, accurate volumetric assessment requires standard operating procedures that include the know-how specific for the modality, acquisition parameters, and suitable training of the staff [5]. For this reason, strict standardization of acquisition and measurement is being undertaken for manual hippocampal volumetry by a European Alzheimer’s Disease Consortium (EADC)-ADNI consortium under the auspices of the Alzheimer’s Association [17,44]. The EADC-ADNI standard operating procedures will be used to validate automated algorithms, such as FreeSurfer and Learning Embeddings Atlas Propagation. Other brain regions are under active investigation; for example, the entorhinal cortex showed performance similar to hippocampus using the Quarc analysis software (Quarc, Wedemark, Germany) [45]. According to this study, when these markers were used as primary endpoints, a sample size of about 100 subjects with AD would be sufficient to detect a 25% reduction of annual atrophy rate produced by a putative novel treatment [45]. This is at least threefold less than the sample size needed when using clinical scales such as the Alzheimer’s Disease Assessment Scale-cognitive subscale as endpoints.

#### Vascular magnetic resonance imaging

The modality of T2-weighted/fluid inversion recovery is particularly useful to identify vasogenic edema and microhemorrhages, defined as the amyloid-related imaging abnormalities (ARIAs) in AD research [19,43]. Regulatory authorities require their use in clinical trials implementing the β-amyloid-lowering drugs for safety reasons. For example, in two bapimezumab phase II trials, ARIAs occurred in 17% of patients with AD, 78% of whom did not report clinical correlates [19]. The occurrence of ARIAs seems to depend on the dose of the β-amyloid-lowering drug and on the presence of the apolipoprotein E4ϵ genotype, which is characterized by a significantly higher β-amyloid burden [19,41]. A rating scale was recently proposed [46].

### Diffusion tensor imaging magnetic resonance imaging

Through the assessment of the random motion of water molecules in the tissue, diffusion tensor imaging (DTI) has provided new tools to the study of white matter alterations in AD brain mostly by assessing two parameters: the fractal anisotropy (FA) and the mean diffusivity (MD). FA is considered a marker of axonal integrity and myelinization, whereas MD represents the overall cellular integrity [47]. The combined use of DTI and volumetric MRI in the cingulated cortex was shown to increase the imaging-based classification of AD cases versus control with up to 91% accuracy [21]. Initially, DTI has been perceived as a technique with poor reproducibility and site/scanner dependency. Recent multi-center trials in Huntington’s disease and AD seem to contradict this preoccupation, suggesting a low center-dependent bias and a real possible use as a surrogate endpoint [22,23].

### Functional magnetic resonance imaging

fMRI can be defined as a technology that provides statistical maps of brain activation [6,7]. Brain activation is indirectly obtained by measuring changes of regional microcirculation produced by local neural activity triggered by external stimuli, behavioral performance, or neuroactive drugs. Two kinds of magnetic resonance signals are generally measured: the blood oxygenation level-dependent (BOLD) and the arterial spin labeling (ASL) signals [7,48].

#### Activation task functional magnetic resonance imaging procedures

Most brain activation maps obtained in patients with AD have been generated during memory tasks and using BOLD, showing reduced activity in hippocampus/medial temporal lobe and increased activity in prefrontal cortex while encoding new information [30,31]. Similar abnormalities, in particular decreases in the medial temporal lobe, were observed in subjects with prodromal AD/mild cognitive impairment (MCI) [48]. Intriguingly, increases were described in the early phase of the disease [32] and in asymptomatic individuals with genetic risk for AD [24]. This hyper-activation was interpreted as a possible compensatory mechanism at work within the networks whose structures were impaired, a pathologic marker of impending neural failure [9]. Whereas data on longitudinal test-retest were provided in single-center studies [33], fMRI activation tasks were very rarely used in multi-center studies, highlighting the need for further validation and standardization.

Logistically, the typical fMRI activation task is quite complex, requiring audio/visual support for stimuli presentation (for example, goggles) and performance recording (for example, joysticks), expert personnel, calibration procedures for each tasks, and proven invariance to repeated exposure (often not studied), therefore making their implementation difficult in multi-center trials or in routine use as a diagnostic in the community.

#### Task-free functional magnetic resonance imaging procedures

More recently, resting-state fMRI (rs-fMRI) was introduced to collect signals while the subject is not performing any particular tasks [6,7]. This task-free approach is ideal in poorly collaborative subjects, such as severely demented patients, and does not require a specialized staff. rs-fMRI is based on the collection of time series of whole-brain MRI signal fluctuations (generally BOLD) measured in each brain voxel while the subject is at rest [6,7]. The similarity between time series in different brain areas can be assessed mathematically as correlation matrices, indicating functional connectivity [29]. Consistent patterns of connectivity at resting state were reported by several authors, evaluated in test-retest protocol, and related to specific electrophysiological signatures in topologically restricted brain areas, exemplified by the 'default mode’ network (DMN) [34]. In AD, impaired DMN has been reported, including longitudinal data that showed a correlation with clinical symptom worsening [6]. Interestingly, rs-fMRI studies in non-demented adults carrying a familial AD gene mutation showed hyper-connectivity in the dorsolateral prefrontal cortex and decreased connectivity in the precuneus [26,27]. rs-fMRI is also a stronger classifier than memory task-associated fMRI when used in non-demented adults carrying a familial AD mutation [27]. Interestingly, changes of connectivity in the DMN of subjects with AD were observed following chronic treatment with memantine [49] and donepezil [25].

#### Arterial spin labeling

In the last few years, ASL has attracted the attention of various research groups [7]. ASL MRI was validated versus perfusion PET showing convergent information about regional cerebral blood flow of diagnostic relevance, with higher resolution and no exposure to radioactive tracers [25,28]. Evidence of hypoperfusion in AD and prodromal AD/MCI conditions was confirmed and extended to a series of other brain areas, and the initial evidence is that ASL may have properties of diagnostics and markers of disease severity [20,25,50]. ASL measurements appear less variable than BOLD in test-retest trials in a single center. Unfortunately, to date, it cannot be easily implemented in multi-center trials since different vendors of MRI scanners have implemented different sequences of acquisition, therefore hindering comparison among sites with different MRI scanners.

## Conclusions and future directions

There is a role for MRI as a biomarker in clinical trials aimed at the development of new treatment for AD. However, a series of challenges is still looming, in particular for fMRI. Among them, we have identified reliability and repeatability for specific tests, standardization across multiple sites, development of validated automated quantification tools, implementation of multiple modalities (for example, merging PET and rs-fMRI), cost optimization, and selection of the most accurate and efficient diagnostic combination of markers, including fluid-based biomarker modalities. Most of these challenges are tackled in the context of large consortia involving collaborations between academia and industry as well as in some contract research organizations and technology-oriented academic centers. The delivery of this research is of relevance for building confidence in imaging biomarkers: at present, with the exception of safety assessment of ARIA signals, the implementation of MRI in pharmaceutical clinical trials is still seen as exploratory rather than decision-making.

## Abbreviations

AD: Alzheimer’s disease; ADNI: Alzheimer Disease Neuroimaging Initiative; ARIA: Amyloid-related imaging abnormality; ASL: Arterial spin labeling; BOLD: Blood oxygen level-dependent; DMN: Default mode network; DTI: Diffusion tensor imaging; EADC: European Alzheimer’s Disease Consortium; EMA: European Medicines Agency; FA: Fractal anisotropy; FDG: Fluoro-2-deoxy-D-glucose; fMRI: Functional magnetic resonance imaging; MCI: Mild cognitive impairment; MD: Mean diffusivity; MRI: Magnetic resonance imaging; NCE: Novel chemical entity; NFT: Neurofibrillary tangle; PET: Positron emission tomography; rs-fMRI: Resting-state functional magnetic resonance imaging.

## Competing interests

AL is an employee of GlaxoSmithKline (Brentford, UK). MES is an employee of Janssen Pharmaceutica (Beerse, Belgium). AJ is an employee of Atlantic Biomarkers, LLC (Gainesville, FL, USA). EMP is an employee of F. Hoffmann-La Roche AG (Basel, Switzerland). DH is an employee of IXICO (London, UK). GBF has served on advisory boards for Eli Lilly and Company (Indianapolis, IN, USA), Bristol-Myers Squibb (New York, NY, USA), Bayer (Leverkusen, Germany), Lundbeck (Copenhagen, Denmark), Elan (Dublin, Ireland), AstraZeneca (London, UK), Pfizer Inc (New York, NY, USA), TauRx (Aberdeen, UK), Wyeth (Philadelphia, PA, USA), GE (Fairfield, CT, USA), and Baxter (Deerfield, IL, USA); is a member of the editorial boards of *Lancet Neurology*, *Aging Clinical & Experimental Research*, *Alzheimer’s Diseases & Associated Disorders*, and *Neurodegenerative Diseases*; is the imaging section editor of *Neurobiology of Aging*; has received grants from Wyeth International, Lilly International, Lundbeck Italia, GE International, Avid/Lilly, Roche, and the Alzheimer’s Association; and in the past 2 years has received lecture fees to speak at the invitation of Lundbeck. WZP has served on advisory boards for Amgen (Thousand Oaks, CA, USA), Allelix (Mississauga, ON, Canada), Ironwood (Cambridge, MA, USA), Eli Lilly and Company, Taisho (Tokyo, Japan), Takeda (Osaka, Japan), and Teva (Petah Tikva, Israel) in the last 12 months. SM declares that she has no competing interests.
